# Supplementary material for: WordCommentsAnalyzer: A windows software tool for qualitative research
Source: F1000Res. 2018 Sep 4;7:536. Originally published 2018 May 3. [Version 2] doi: 10.12688/f1000research.14819.2 (PMC6137411; doi:10.12688/f1000research.14819.2)
Supplement: Supplementary file 3 [file f1000research-7-17614-s0002.tgz › b2a79496-4ada-47c4-9682-a99ce867836d.zip!/Eight Iranian Health Journals/Journal of Research in Rehabilitation Sciences _52.docx]

© Journal of Research in Rehabilitation Sciences . This work is licensed under CC BY-NC 4.0 (https://creativecommons.org/licenses/by-nc/4.0/).

Volume & Issue: 1386

Title: بررسی اثر تغییرات دوره‌ای فشار مثبت و منفی در ترمیم زخم پای دیابتی

http://jrrs.mui.ac.ir/index.php/jrrs/article/view/83

مقدمه: مطالعات جديد نشان مي‌دهند درمان با فشار و مکش متناوب (VCT) (vacuum compression therapy) با بهبود گردش خون شرياني و وريدي مي‌تواند در بيماران مبتلا به لنگش متناوب و احتمالاً زخمهاي ناشي از آنژيوپاتي مفيد واقع شود. با توجه به اين نكته، مطالعه حاضر براي بررسي كارايي VCT در درمان زخمهاي ناشي از آنژيوپاتي در بيماران ديابتي طراحي و اجرا شده است. مواد و روشها: در يك كارآزمائي باليني، 16 بيمار به مدت 4 هفته تحت درمان با VCT و ساير درمانهاي معمول زخمهاي ديابتي قرار گرفتند و 12 بيمار نيز در گروه شاهد فقط درمانهاي معمول را دريافت كردند. كاهش اندازه سطح و عمق زخم در دو گروه پس از پايان دورهء درمان مورد مقايسه قرار گرفت. نتايج: ميانگين اندازه سطح  زخم در گروه مداخله  34/0 ± 26/1 سانتيمتر مربع كاهش و در گروه شاهد28/0 ± 44/0 سانتيمتر مربع افزايش يافته بود (0000/0 = p). عمق زخم در پايان دوره درمان در گروه VCT به طور متوسط 63/0 ± 4 ميليمتركاهش يافته بود، در حاليكه در گروه شاهد21/0 ± 33/0  ميلي‌متر افزايش نشان مي­داد (0000/0 = p). بحث و نتیجه‌گیری:VCT مي‌تواند به عنوان يك روش موثر در درمان زخم پاي ديابتي مورد استفاده واقع شود. البته اين روش نيز موارد منع استفاده و محدوديتهاي خاص خود را دارد كه در به كارگيري اين روش بايد به آنها توجه داشت.

Title: بررسي تأثير بي‌حسي انتخابي موقتي همراه با بازآموزي حسي برروي بهبود حساسيت‌پذيري دست بعد از ترميم اعصاب مديان/اولنار

http://jrrs.mui.ac.ir/index.php/jrrs/article/view/84

مقدمه: نتایج حسی، پس­از ترمیم عصب در بزرگسالان، عموماً ضعیف می­باشد. ما فرض نمودیم آوران‌زدائی ساعد نتایج حسی را ازطریق افزایش نمایش کورتیکال دست بهبود می­بخشد. مواد و روش­ها: یک مطالعه آینده­نگر تصادفی double-blind انجام شد. در طی دوره دو هفته­ای یک کرم بی­حسی موضعی        (Lidocaine-PTC) در گروه مورد (تعداد=6)، یا placebo در گروه کنترل (تعداد=7)، مکررا (4بار) بر روی وجه فلکسور ساعد دست آسیب‌دیده مورد استفاده قرار گرفت. برنامه بازآموزی حسی برای هر دو گروه یک ساعت پس از استعمال پماد انجام شد. ارزیابی حسی در فواصل منظم، قبل از مداخله،پس­از آخرین مداخله (پایان جلسه چهارم) و 4 هفته (follow-up) پس­از آخرین کاربرد پماد Lidocaine-PTC / placebo انجام شد. نتایج: گروه Lidocaine-PTC بهبودی قابل­توجهی در مقایسه با گروه placebo از نظر درک لمس/فشار (005/0P=) و TPD ثابت (005/0P=) پس­ از 6 هفته کسب نمودند. بحث و نتیجه­گیری: بی­حسی موضعی ناحیه ساعد آسیب­دیده در ترکیب با بازآموزی حسی می­تواند نتایج حسی را پس­از ترمیم عصب ارتقاء دهد.

Title: بررسي تأثير بار خارجي و وضعيت نته بر انحناي كمر در حالت استاتيك

http://jrrs.mui.ac.ir/index.php/jrrs/article/view/85

مقدمه: مطالعات اپيدميولوژيك نشان داده است كه كمردرد به دنبال انجام بلند کردن اجسام، یکی از اختلالات مهم در سيستم عضلاني- اسكلتي مي‌باشد و عامل مهمي در ايجاد ناتواني عملكردي براي بيمار و ضررهاي سنگين اقتصادي است. انحناي كمر يكي از مهمترين مشخصات پاسچر و حركت بدن مي‌باشد كه جهت فهم بهتر مشكلات كمر درد با اهميت است. چنانچه تغيير انحناي كمر در طي حركات، ميزان استرس‌هاي وارد روي ستون فقرات را متأثر مي‌كند. تطابق هندسی ستون فقرات كه مستقيماً وابسته به مقدار انحناء است از عوامل مؤثر در کاهش دادن بارهاي فشاري روي ستون فقرات مي‌باشد. مطالعات استاتيكي حفظ بار نشان داده است، در حالتي كه فرد وزنه‌اي در دستان خود داشته باشد، بهترين انحنای کمر بستگي به مقدار بار و زاويه خميدگي در تنه دارد. هدف از اين مطالعه بررسي تأثير بار خارجي و وضعيت تنه برميزان انحنای کمر در حالت استاتيك است. مواد و روشها: اين مطالعه بر روي‌10‌زن سالم بدون سابقه بيماريهاي قلبي- عروقي يا اسكلتي-‌ عضلاني، انجام شد. با استفاده از دو حسگر شيب سنج الکتروليتی ميزان انحنای کمر افراد در شش فعاليت استاتيكی با سه سطح بار خارجي (صفر، شش ودوازده كيلوگرم) و دو وضعيت تنه (نوترال و خميده‌ي 30 درجه) مورد بررسي قرار گرفت. نتایج: نتايج نشان داد كه وضعيت خميده تنه(خميده‌ي 30 درجه) و بار خارجی(شش ودوازده كيلوگرم) موجب كاهش لوردوز کمر مي‌گردد(05/ 0P <). بحث و نتیجه‌گیری: صاف شدن لوردوز كمر هنگام حفظ بار، نياز به فعاليت عضلاني و آسيب به بافت‌هاي اطراف را  كاهش مي‌دهد. افزايش کيفوز كمر در اثر خميدگي تنه يك عامل حمايت كننده از ستون فقرات كمر در برابر استرس‌هاي فشاري است.

Title: تعيين ميزان تأثير استرپ‌هاي اينفراپاتلار و چوپات در قدرت و دامنه حركتي زانو در بيماران مبتلا به سندرم درد قدامي زانو

http://jrrs.mui.ac.ir/index.php/jrrs/article/view/86

مقدمه: سندرم درد قدامی زانو (Anterior Knee Pain Syndrome) یکی از شایعترین مشکلات مفصل زانو است بطوریکه 31-23% از مراجعه کنندگان به درمانگاههای ارتوپدی را این افراد تشکیل میدهند (1). پايداري زانو بستگي به ليگامانهاي اصلي آنو عضله چهار سر ران دارد. اهميت عضله چهار سر را نمي‌توان ناديده گرفت، بطوريكه عضله چهارسر قدرتمند قادر به كنترل مؤثر عملكرد زانوست و مي­تواند عليرغم شلي قابل توجه ليگامانهاي ذكر شده، پايداري زانو را حفظ كند (2). يكي از متداولترين روشهاي درماني افراد مبتلا به سندرم درد قدامي زانو استفاده از حمايت كننده­هاي خارجي زانو و ارتزها مي‌باشد (2،3،4). مواد و روش­ها:  تعداد 30 نفر افراد مبتلا به درد قدامی زانو با انجام مراحل خاص غربالگری وارد مطالعه شدند. افراد را بطور تصادفي به دو گروه 15 نفره تقسيم كرده و تحت درمان ارتزي قرار گرفتند. میزان قدرت اکستنشن Extension)) با استفاده از داینامومتر دیجیتال، دامنه حرکتی با استفاده از گونیامتر و میزان درد ازطریق آزمون سنجش درد ديداري (Visual Analogue Scale (VAS)) در قبل، بلافاصله بعد و دو هفته بعد از استفاده از استرپ­های چوپات و اینفراپاتلار اندازه­گیری شد. داده­ها با استفاده از آزمون آنالیزواریانس با تکرار مشاهدات مورد ارزیابی قرار گرفت. میزان قدرت اکستنشن، دامنه حرکتی و میزان درد تفاوت معنی داری در سه مرحله آزمون از خود نشان داد. بحث و نتیجه­گیری: استفاده از استرپ­های چوپات و اینفراپاتلار میتواند باعث افزایش قدرت اکستنشن، کنترل دامنه حرکتی فلکشن(Flexion) و کاهش درد در افراد مبتلا به سندرم درد قدامی زانو شود.

Title: مقايسه فاكتورهاي تنفسي در بيماران مبتلا به ندول تار صوتي و افراد سالم

http://jrrs.mui.ac.ir/index.php/jrrs/article/view/87

مقدمه: گفتار مهمترين ابزار برقراري ارتباط بين انسانهاست و حاصل عملكرد هماهنگ سيستمهاي تنفسي، آواسازي، تشديدي و توليدي مي­باشد. امروزه افزايش فشارهاي ناشي از زندگي اجتماعي و وجود محيطهاي نامناسب مملو از مواد آلاينده، انواع مختلفي از اختلالات صوتي را موجب شده ­است. در بين اين اختلالات، ندول تارصوتي از شيوع بيشتري برخوردار است. با توجه به اين امر كه هواي مورد نياز براي ارتعاش تارهاي صوتي توسط سيستم تنفسي تامين مي­شود و نامناسب بودن فاكتورهاي تنفسي در بيماران مبتلا به ندول تارصوتي همواره مشاهده مي­گردد، اين پژوهش به بررسي انواع مختلف فاكتورهاي تنفسي در بيماران مبتلا به ندول تارصوتي و مقايسه آن با افراد سالم پرداخته ­است. مواد و روشها: اين پژوهش از نوع مقطعي و توصيفي- تحليلي است. جامعه مورد مطالعه21 نفر، شامل14 بيمار مبتلا به اختلال صوت ارجاع شده توسط متخصص گوش و حلق بيني و 7 نفر سالم مي‌باشند. با استفاده ازدستگاههاي PCLX، LX-Strobe، ST1 Dysphonia حجم حياتي، حجم جاري، حجم تنفس در حين آواسازي بيماران و افراد سالم اندازه­گيري شد و ازآزمونهاي فيشر و آزمون من – ويتني براي تجزيه و تحليل نتايج استفاده گرديد. نتايج: نشان مي­دهد كه بيماران مبتلا به ندول تارصوتي از نظر حجم حياتي، حجم جاري، حجم تنفسي در حين آواسازي با فركانسهاي زير، عادتي و بم، و همچنین زمان آواسازي با فركانسهاي زير، عادتي و بم داراي تفاوت معناداري با افراد سالم مي­باشند و ارتباط بين ندول تارصوتي و پيشينة خانوادگي معنادار نبوده ­است. بحث و نتیجه­گیری: حجم ششها در افراد مبتلا به ندول تارصوتي كمتر از افراد سالم نيست. تنش زياد در عضلات تنفسي در طي صحبت كردن به این بيماران اجازه نمي­دهد كه از حجم ششهاي خود به طور مناسب استفاده ­كنند و اين امر موجب كاهش حجم تنفسي و در نتيجه اختلال سيستم آواسازي مي­گردد.

Title: تأثير هشت هفته حركات اصلاحي بر لوردوز كمري

http://jrrs.mui.ac.ir/index.php/jrrs/article/view/88

مقدمه: حركات اصلاحي يا ورزش‌هاي اصلاحي شاخه‌اي از علوم كاربردي در سطح جامعه بويژه در مواردي است كه مشتمل بر اهداف عمده شناسائي، آموزش، پيشگيري، بهبود و اصلاح ناهنجاري‌هاي بدن و عادات غلط حركتي افراد است.  هدف از اين مطالعه تأثير انجام هشت هفته حركات اصلاحي بر عارضه  لوردوز كمري دانشجويان دختر22-19 ساله دانشگاه اصفهان مي‌باشد.  مواد و روشها: جامعه آماري تحقيق حاضر شامل کليه دانشجويان دختر مبتلا به لوردوز کمري (300 نفر) دانشگاه اصفهان مي‌باشد. تعداد 44 نفر از دانشجويان مبتلا به قوس کمري افزايش يافته با ميانگين سن(68/1±50/21)، قد(83/5 ± 64/160) و وزن (89/7 ± 42/55) به عنوان نمونه آماري انتخاب شدند و به طور تصادفي در دو گروه 22 نفري آزمون و کنترل قرار داده شدند. اطلاعات مورد نياز از طريق صفحه شطرنجي، خط‌كش منعطف (سنجش قوس كمر)، قد و وزن‌سنج ديجيتال، متر نواري (سنجش ميران انعطاف‌پذيري عضلات كمر)، گونيا متر (سنجش ميزان كوتاهي عضلات خم‌كننده‌ي ران) جمع‌آوري شدند. براي گروه آزمون هشت هفته، هفته‌اي 3 جلسه و هر جلسه60 دقيقه با شدت 85 – 60 درصد (حداکثر ضربان قلب) تمرينات اصلاحي اعمال شد. در آغاز و پايان دوره از آزمودني‌ها پيش و پس آزمون به عمل آمد. اطلاعات جمع‌آوري شده توسط روش آماري t مستقل و t زوج شده مورد تجزيه و تحليل آماري قرار گرفت. كليه تحليل‌ها در سطح معناداري 5 ./. انجام گرديد.  نتايج: كاهش معناداري در زاويه لوردوز كمري (005/0=P) افزايش معني‌داري در انعطاف عضلات اکستنسور ستون فقرات (000/0=P) و افزايش معناداري در قدرت و استقامت عضلات شكم (000/‌0=‌P) ديده شد ولي در انعطاف‌پذيري عضلات فلکسور ران در پس آزمون (24/0=P) افزايش مشاهده شده از نظر آماري معنادار نبود. بحث و نتيجه‌گيري: بعد از انجام هشت هفته حركات اصلاحي درجه ي قوس كمر به صورت معنا‌داري كاهش يافت. در ميزان قدرت و استقامت عضلات شكم، انعطاف‌پذيري عضلات اکستنسور ستون فقرات افزايش معنا‌داري مشاهده گرديد. ولي در افزايش انعطاف‌پذيري عضلات فلکسور ران اين نتيجه مشاهده نشد. به طور كلي برنامه اصلاحي ارائه شده در دستيابي به اهداف خود موفق بود.

Title: بررسی الگوی فعالیت فیزیکی پرستاران بیمارستانهای تأمین اجتماعی استان اصفهان

http://jrrs.mui.ac.ir/index.php/jrrs/article/view/89

مقدمه: با توجه به افزايش شيوع بيماريهاي غيرواگير كه كم تحركي از عوامل زمينه‌ساز آنهاست مسئولين بهداشتي درصدد تشويق افراد به انجام فعاليت فيزيكي بيشتر مي‌باشند. پرستاران از جمله افرادي هستند كه بواسطه شغل خود در معرض ابتلا به بيماريهاي غيرواگير از جمله مشكلات قلبي عروقي و عضلاني اسكلتي و روحي- رواني قرار دارند. هدف اين مطالعه بررسي الگوي فعالیت فيزيكي پرستاران شاغل در بيمارستانهاي وابسته به سازمان تامين اجتماعي در استان اصفهان مي‌باشد. مواد و روشها: در اين مطالعه مقطعي توصيفي تحليلي 82 پرستار شاغل در بيمارستانهاي تامين اجتماعي استان اصفهان بررسی شدند. ابزار جمع‌آوري اطلاعات پرسشنامه استاندارد شده فعاليت فيزيكي 7 روزه IPAQ‌(International Physical Activity Questionnaire) مي‌باشد.  اين پرسشنامه داراي 5 قسمت است (فعاليت فيزيكي مربوط به شغل، اوقات فراغت، منزل و جابجايي و فعاليتهاي نشسته) كه در هر مورد تعداد روزهايي كه فرد حداقل به مدت 10 دقيقه به آن فعاليت پرداخته و مدت زماني كه در يك روز به آن فعاليت پرداخته بر حسب ساعت و دقيقه سوال مي‌شود. نتایج: ميانگين فعاليت فيزيكي شغلي و جابه جايي در دو جنس تفاوت معني داري نداشت ولي ميانگين فعاليت فيزيكي در منزل در زنان نسبت به مردان به طور معني‌داري بيشتر بود. ميانگين فعاليت فيزيكي اوقات فراغت مردان نسبت به زنان به طور معني داري بيشتر بود. مقايسه ميزان فعاليت فيزیكي در 3 بيمارستان، نتايج تفاوت معني‌داري را نشان نداد. بحث و نتيجه‌گيري: با توجه به اينكه درصد زيادي از جامعه پرستاري از نظر فيزيكي فعاليت كافي ندارند تاكيد بر انجام فعاليت فيزيكي به ويژه در اوقات فراغت به منظور جابه جايي و به ويژه در پرسنل بهداشتي از جمله پرستاران در كاهش اين عوامل خطر و كنترل و پيشگيري از اين بيماريها تاثير به سزايي دارد.

Title: ساختار سازه‌ای هر یک از واکه‌های زبان فارسی در دانش‌آموزان 15 تا 18 ساله عادی و مبتلا به افت شنوایی متوسط و شدید شهر اصفهان در مقطع دبیرستان

http://jrrs.mui.ac.ir/index.php/jrrs/article/view/90

مقدمه: یکی از مهمترین ویژگیهای فیزیکی آواهای گفتار، ‌ساختار سازه‌ای است. اهمیت ساختار سازه‌ای به گونه‌ای است که روابط میان سه سازه اول از اجزای اصلی تشخیص نوع واکه توسط شنونده است. تشخیص غیرطبیعی بودن واکه‌های فرد مبتلا به افت شنوایی بر پایه ادراک درمانگر از صدای او نیست بلکه اندازه‌گیری میزان فرکانس او به کمک دستگاه تشخیص را دقیق‌تر می‌کند. هدف از این مطالعه تعیین و مقایسۀ ‌ساختار سازه‌ای واکه‌های زبان فارسی در دانش آموزان عادی و مبتلا به افت شنوایی متوسط و شدید محدودۀ سنی 18-15 سال مقطع دبيرستان می‌باشد. مواد و روشها: این پژوهش توصیفی- تحلیلی و به صورت مقطعی روی 64 نفر دانش آموز از دبیرستانهای عادی و دبیرستانهای مربوط به  دانش آموزان مبتلا به افت شنوایی شهر اصفهان انجام گرفت. میانگین F2 , F1 و ميانگين F2/F1 وF3/F1 در هر 6 واکه‌ی زبان فارسی در بین سه گروه دانش‌آموزان عادی و مبتلا به افت شنوایی متوسط و شدید تفاوت معنی‌داری داشت. (05/0 > P‌)، میانگین F3 در تمام واکه‌ها به جزء در واکه /u/ تفاوت معنی‌داری را نشان نداد. نتایج: حاصل از این پژوهش نشان می‌دهد دانش آموزان مبتلا به افت شنوایی متوسط و شديد الگوی نامنظمی از ترتیب قرارگیری واکه‌ها از لحاظ بیشترین و کمترین سازه اول و دوم و سوم را نسبت به افراد عادی نشان می‌دهند و تمایل به جابجایی واکه‌های پیشین با پسین و نزدیکتر کردن محل تولید واکه‌ها به ناحیۀ انتهایی زبان و تولید واکۀ‌ خنثی كه به دلیل وابستگی بیشتر آنها به حس عمقی هنگام تولید صداها است. بحث و نتیجه‌گیری: افراد مبتلا به افت شنوایی (بسته به میزان افت شنوایی) متمایل به تولید مشابه همه واکه‌ها دارند و محل تولید واکه‌ها را به هم نزدیک می‌کنند چون واکه‌های باز را نیم بسته و واکه‌های بسته را نيم باز و واکه‌های پسین را پیشین‌تر و واکه‌های پیشین را پسین‌تر تولید می‌کنند.

Title: بررسی تاثیر درمانی با استفاده از Swiss Ball در بهبود تعادل افراد مبتلا به پارکینسون

http://jrrs.mui.ac.ir/index.php/jrrs/article/view/91

مقدمه: بيماري پاركينسون يك بيماري دژنراتيو و پيشرونده هسته­هاي قاعده­اي مغز است و چهار علامت اصلي آن برادي كينزي، ترمور، رژيديتي و بي­ثباتي پاسچرال است، كه بي­ثباتي پاسچرال از بزرگترين عوامل افتادن در اين افراد مي­باشد . عليرغم اثبات نقش تمرين درماني در كاهش عوارض ثانويه بي­حركتي در اين افراد، شواهد كافي براي بيان تاثير اين تمرينات وجود ندارد كه اين مطالعه در جهت رفع اين خلا انجام گرفته است. مواد و روشها: اين مطالعه از نوع آينده نگر، شبه تجربي و يك سويه كور بوده كه در سال­هاي 86-85 در كلينيكهاي فيزيوتراپي وابسته به دانشكده توانبخشي اصفهان انجام گرفته است. از ميان بيماران پاركينسوني مراجعه كننده به بيمارستان الزهرا با مشورت متخصص آمار 23 نفر با داشتن شرايط ويژه (75/7  ± 43/67  = سن ،  44/8 ± 74/164 = قد ،85/7 ± 61/65=وزن) به صورت غير تصادفي انتخاب گرديدند و 12 جلسه (هفته اي 3 جلسه و هر جلسه حدود يك ساعت) تحت تمريناتي با استفاده از Swiss Ball و با نظارت كامل تراپيست قرار گرفتند. قبل و بعد از درمان و همچنين بعد از يك ماه follow up  جهت بررسي بالانس اين بيماران از آزمون Berg Balance Scale و جهت بررسي افسردگي اين بيماران از پرسشنامه Beck استفاده گرديد. همچنين براي تجزيه و تحليل داده­ها از نرم افزار SPSS و آزمون t زوج شده استفاده شد. نتايج: در ارزيابي بالانس بين قبل و بعد از درمان (00/0 =P) وبين قبل از درمان و بعد از يك ماهfollow up  (00/0 =P) تفاوت معني‌داري حاصل گرديد. در ارزيابي بالانس بين بعد از درمان و يك ماه follow up تفاوت معني داري حاصل نگرديد (141/.=P). در ارزيابي افسردگي بين قبل و بعد از درمان (00/0=P) و بين قبل از درمان و بعد از يك ماهfollow up (00/0=P) تفاوت معني­داري حاصل گرديد. در ارزيابي افسردگي بين بعد از درمان و يك ماه follow up تفاوت معني­داري حاصل نگرديد.(56/.=P) بحث و نتيجه‌گيري: با توجه به نتايج حاصل مي­توان چنين نتيجه گرفت كه استفاده از تمرين درماني با استفاده از Swiss Ball باعث بهبود بالانس و افسردگي حداقل براي يك ماه در اين بيماران خواهد شد. اما بايد توجه داشت كه با توجه به پيشرونده بودن اين بيماري اگر اين تمرينات به صورت روزانه تداوم نيابد، ايجاد مجدد مشكلات بالانس و افسردگي اجتناب ناپذير خواهد بود.

Title: اختلال در فرایند رمز گشایی زبانی در مغز افراد زبان پریش دو زبانه

http://jrrs.mui.ac.ir/index.php/jrrs/article/view/92

مقدمه: در مقاله‌ی حاضر پس از معرفی بعضی از دیدگاه‌های موجود درباره‌ی چگونگی عملکرد مغز در افراد دو زبانه‌ی زبان پریش با توجه به دیدگاه‌های علوم اعصاب و عصب‌شناسی زبان، تبیین نویسنده با توجه به یافته‌های حاصل از تکنیکهای جدید تصویربرداری  مغزی و یافته‌های بدست آمده از بررسی‌های افراد زبان پریش و الگوهای مختلف بهبودی بیان گردیده است. بر اساس شواهد و نظام عملکرد مغزی می‌توان چنین تصور نمود که بر طرف شدن اختلالات زبانی با توجه به خود- سازماندهی مغزی، می‌تواند تحت تاثیر فرایندهای هماهنگ سازی تولید و درک زبانها با ساختاری مغز و با دیدی ظریفتر ناشی از برطرف شدن اختلال در سامانه رمزگشایی زبانی باشد.
